# Regulation of multiple tip formation by caffeine in cellular slime molds

**DOI:** 10.1186/1471-213X-12-26

**Published:** 2012-08-28

**Authors:** Pundrik Jaiswal, Shashi Prakash Singh, Prasad Aiyar, Rakhil Akkali, Ramamurthy Baskar

**Affiliations:** 1 Department of Biotechnology, Indian Institute of Technology-Madras, Chennai 600036, India

## Abstract

**Background:**

The multicellular slug in *Dictyostelium* has a single tip that acts as an organising centre patterning the rest of the slug. High adenosine levels at the tip are believed to be responsible for this tip dominance and the adenosine antagonist, caffeine overrides this dominance promoting multiple tip formation.

**Results:**

Caffeine induced multiple tip effect is conserved in all the Dictyostelids tested. Two key components of cAMP relay namely, cAMP phosphodiesterase (Pde4) and adenyl cyclase-A (AcaA) levels get reduced during secondary tip formation in *Dictyostelium discoideum*. Pharmacological inhibition of cAMP phosphodiesterase also resulted in multiple tips. Caffeine reduces cAMP levels by 16.4, 2.34, 4.71 and 6.30 folds, respectively in *D. discoideum, D. aureostipes, D. minutum* and *Polysphondylium pallidum*. We propose that altered cAMP levels, perturbed cAMP gradient and impaired signalling may be the critical factors for the origin of multiple tips in other Dictyostelids as well. In the presence of caffeine, slug cell movement gets impaired and restricted. The cell type specific markers, ecmA (prestalk) and pspA (prespore) cells are not equally contributing during additional tip formation. During additional tip emergence, prespore cells transdifferentiate to compensate the loss of prestalk cells.

**Conclusion:**

Caffeine decreases adenyl cyclase–A (AcaA) levels and as a consequence low cAMP is synthesised altering the gradient. Further if cAMP phosphodiesterase (Pde4) levels go down in the presence of caffeine, the cAMP gradient breaks down. When there is no cAMP gradient, directional movement is inhibited and might favour re-differentiation of prespore to prestalk cells.

## Background

Cellular slime molds are unicellular, free living soil amoebae alternating its life cycle between growth and multicellular development [1]. As amoebae, they prey on bacteria and multiply until all the food is exhausted. At the onset of starvation, the amoebae secrete chemoattractants to communicate with each other enabling them to form a multicellular aggregate. The aggregates transform to a motile slug which later culminates to a fruiting body with a dead stalk holding a mass of dormant spores. *D. discoideum* slug consists of two prominent cell types: the anterior prestalk cells and the posterior prespore cells [2]. Four morphogenetic regulators viz., cAMP, adenosine, ammonia (NH_3_) and differentiation inducing factor (DIF) coordinate and regulate cell fate and cell type proportioning during development in slime molds [3-6].

Cellular slime molds are grouped in 4 distinct evolutionary lineages based on the small subunit ribosomal DNA (SSU) rDNA and α-tubulin amino acid sequences [7]. Group 1 species-*D. aureostipes*, Group 2 species-*Polysphondylium pallidum*, Group 3 species-*D. minutum* and Group 4 species-*D. discoideum* makes use of, an unknown compound, glorin, folic acid and cAMP respectively, as chemoattractants for their aggregation [8-13]. Caffeine is known to induce multiple tip formation in *D. discoideum* and it is not clear if multiple tip formation induced by caffeine is common to other cellular slime molds.

Tip dominance is one of the crucial steps in slug volume regulation during morphogenesis in cellular slime molds [14]. The single slug tip, like an organiser of the metazoan embryo regulates multicellular development [15]. The tip of the slug acts as a pacemaker [16] and secretes cAMP signals periodically with a propagation speed of 200 μm/min [17]. The cell movement within the slug is directed and move with an average period of 3 minutes [17]. The cAMP waves are initiated at the slug tip and propagate towards the back of the slug [16]. Because of the primary tip dominance, additional tip formation is repressed [14], a phenomenon called tip inhibition and adenosine plays a crucial role in this process by inhibiting new tip formation [14]. The mechanism of multiple tip formation is not well understood but it is believed that cAMP relay might regulate this process [17]. Caffeine is known to inhibit the oscillatory cAMP relay [17,18] and removes tip inhibition by reducing the amplitude and oscillation frequency of cAMP signals [17]. Caffeine, the antagonist of adenosine favours tip activation inducing multiple tip formation [17,19].

The cAMP signal strength and its relay are regulated by the activity of adenyl cyclases (AcaA), cAMP phosphodiesterase (PdsA and Pde4) and cAMP phosphodiesterase inhibitor (PDI) [20-24]. cAMP upon binding to its receptors (cAR1) activates adenyl cyclase to catalyze the conversion of ATP into cAMP [23,24]. The secreted cAMP gets degraded by PdsA into 5’AMP which is negatively regulated by PDI [20,23]. The intracellular cAMP levels are governed by another cAMP phosphodiesterase, RegA. The proteins kinase-A (PKA), the downstream target of intracellular cAMP, upon binding to its regulatory site (PkaR) activates catalytic domain (PkaC) inducing multicellular development [22]. PkaC is known to regulate cAMP relay and genetic lesions in this gene result in defective aggregation [22]. The genes associated with cyclic nucleotide signaling are well conserved across different slime mold species [25].

During secondary tip formation, cells within the slug could possibly sort out or transdifferentiate. Cell sorting is chemotactic to cAMP; prestalk cells sort out by moving towards cAMP source [26]. Cell sorting in *Dictyostelium* is the result of coordinated chemotactic cell movement and cAMP relay kinetics between both the cell types, prestalk and prespore [27]. During tip emergence, cells that move fast and respond strongly to cAMP signalling, collect on the mound tops [27].

Caffeine is an antagonist of adenosine and consist of a purine ring and three methyl groups at 1, 3, 7^th^ position of the ring, which is commonly named as 1, 3, 7 trimethyl xanthine. Adenosine, a hydrolysed derivative of cAMP, is synthesised within the slug tip [14]. cAMP levels are regulated by secreted cAMP phosphodiesterase (Pde4) known to hydrolyze cAMP into 5’AMP [20]. AMP further gets degraded into adenosine by 5’ nucleotidase [28].

Here, we show that the multiple tip formation is conserved in all 4 slime mold groups and this effect is not observed when treated with caffeine analogs. The cAMP relay during multiple tip formation was indirectly monitored by checking the expression levels of adenyl cyclase-A (AcaA) and extracellular cAMP phosphodiesterase (PdsA and Pde4). We quantified cAMP levels in slugs with secondary tips based on an enzyme linked immune sorbent assay (ELISA). During caffeine induced multiple tip formation, there is impaired cell movement in slugs leading to spontaneous transdifferentiation. Cell movement within the slugs was monitored by tracking a small fraction of fluorescently labelled cells. Regeneration of ablated prestalk ecmA region in the slug during multiple tip formation suggests transdifferentiation of prespore to prestalk cells.

## Methods

### Cell culture

*Polysphondylium pallidum* PN500 cells were grown on GYP media [29] in the presence of *E. coli B/r*^*-*^ at 22°C with 70% relative humidity [29,30]. All *Dictyostelium* strains except AX2 were grown on SM/5 agar plates with *K. aerogens* at 22°C*.* AX2 cells were grown in axenic HL5 media (28.6 g bacteriological peptone (Oxoid), 15.3 g yeast extract (Oxoid), 18.0 g Maltose (Sigma), 0.641 g Na_2_HPO_4_ (Merck) and 0.49 g KH_2_PO_4_ (Fluka) per litre, pH 6.4) containing antibiotics (200 units/ml penicillin and 200 μg/ml streptomycin sulphate) at 22°C with constant shaking (150 RPM). When there was visible clearing of the bacterial lawns, the plates were washed thrice with ice-cold phosphate buffer (pH 6.4) and cells were harvested at 400 g with 4 minutes of spinning. The cells were spread on non-nutrient agar plates at density of 2 X 10^6^ cells/cm^2^ and incubated in high humid conditions.

### Neutral red staining and multiple tip formation

Amobae stained with 0.06% neutral red was incubated at 22°C for 10 minutes and washed twice with KK_2_ buffer. Neutral red stained amoebae were spread at a density of 2 X 10^6^ cells/cm^2^ on non-nutrient agar plates and allowed to develop as slugs. Fully developed slugs were transferred using a fine needle onto a buffered agar plate having 5 mM caffeine and observed for multiple tip formation.

### Western blot analysis

To check the expression levels of PdsA, slugs with multiple tips were lysed in 200 μl cell lysis buffer (2% SDS, 0.5 M Tris- pH-6.8) containing 1% mercaptoethanol, and the mixture was heated at 95°C for 5 minutes [31]. 20 μl of the cell lysate was electrophoresed in 10% polyacrylamide gels. Equal loading of the protein lysate was checked by Commassie-blue staining running a parallel gel. Anti-PdsA (1:1000-a kind gift from Carole A. Parent, NIH, USA) polyclonal antibody was incubated over night at 4°C. Then, the secondary HRP conjugated antibodies were incubated at room temperature for one hour.

### Quantitative Reverse Transcription-Polymerase Chain Reaction (qRT-PCR)

We performed qRT-PCR to check the expression levels of Pde4-mRNA, PkaC-mRNA, AcaA-mRNA and 5’NT-mRNA in slugs. Slugs were harvested and RNA was isolated using a Qiagen RNeasy mini kit as per manufacturer’s protocols. RNA integrity was checked in 1% formaldehyde agarose gel. cDNA was prepared using Go Script^TM^ Reverse Transcription system (Promega-USA) using random primers. cDNA was mixed with qPCR master mix (Promega-USA) and qRT-PCR was performed using 7500 applied biosystem Real Time-PCR machine. The primer sequences are mentioned in Table 1.

**Table 1:** List of primers used in Real-Time PCR

**Gene**	**Forward primer**	**Reverse primer**
Pde4	GAAGAAGCAACCATTCTCGT	GTTGTTCAGCTACACATCTTGC
ACA	CATTCTAGAGGCGGTATTGGC	GGAGAAAATGTCTGATTTCGCTT
PKAC	AGAACTTTCACCCTTTGTGG	GGATAACCTGCCAACATTTC
5’NT	GATTTTATAGGACGTCAATTTAC	TCCACCGATTGTAATCACACC
IG7	TCCAAGAGGAAGAGGAGAACTGC	TGGGGAGGTCGTTACACCATTC

### β-galactosidase enzyme assay

Slugs were fixed in 3.7% formaldehyde solution in Z-buffer (60 mM Na_2_HPO_4_, 40 mM NaH_2_PO_4_, 10 mM KCl, 1 mM MgSO_4_ and 2 mM MgCl_2_) for 15 minutes. After decanting the fixative, 0.1% NP-40 solution in Z-buffer was added for 15 minutes. Subsequently the plates were washed with Z-buffer and the fixed samples were submerged in freshly prepared staining solution (20 μl of 1 mM X-gal solution in equal volume of 5 mM K3 [Fe (CN_6_)], and 5 mM K4 [Fe (CN_6_)] solution and incubated at 37°C for 45 minutes before observation.

### cAMP quantification

To quantify cAMP levels in the slugs, a cAMP XP^TM^ assay kit (catalog no.4339) was procured from Cell Signaling, USA. This kit contains a cAMP XP^TM^ rabbit mAB coated 96 well plate and HRP linked cAMP, substrate (TMB) and other necessary reagents. The slugs were lysed in a 100 μl of 1X lysis buffer containing 1 mM PMSF (phenyl methyl sulfonyl fluoride) and the lysed sample was incubated in ice for 10 minutes. 50 μl of the test sample and 50 μl of the HRP-linked cAMP solution were added on to the assay plate and was incubated at room temperature for 3 hours on a horizontal orbital shaker. The supernatant was discarded and the wells were washed thrice with 200 μl of 1X wash buffer and thereafter 100 μl of TMB substrate was added to the wells. Subsequently, the plate was incubated at room temperature for 10 minutes and the reaction was terminated by adding 100 μl of stop solution. The absorbance was measured at 450 nm. The cAMP standard curved was used to calculate the absolute amount of cAMP in the test samples.

### Microscopy

Nikon SMZ-1000 stereo zoom microscope with epi-fluorescence optics was used for monitoring and taking the pictures. The fluorescence images were taken using a Nikon 80i eclispse upright microscope.

## Results

### Kinetics of multiple tip formation

**Figure 1:**
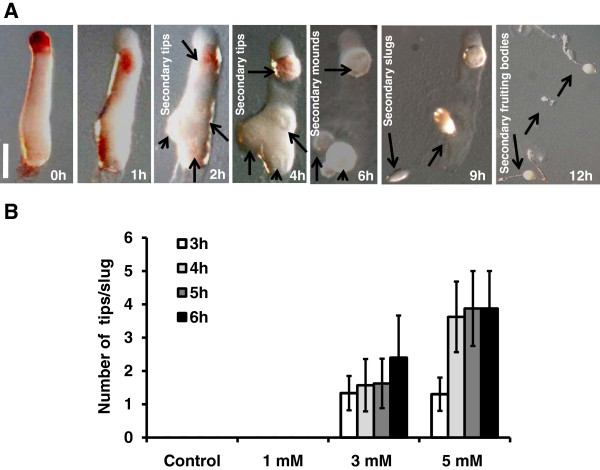
**Effect of caffeine on multiple tip formation in *D. discoideum. *A**) Monitoring multiple tip formation in *D.discoideum*. Freshly harvested cells were stained with neutral red (0.06%) and allowed to form slugs. For getting multiple tip effect, fully developed slugs were transferred with a fine needle onto a non-nutrient agar plate containing 5 mM caffeine as described in the material and methods. Multiple tip formation was monitored by taking pictures at the indicated time points in development. Arrows in Figure 1A represent the emergence of secondary tips at respective stage of development at the mentioned time course. Scale bar = 200 μm. **B**) Graph shows the number of multiple tips in slugs at different concentrations of caffeine at indicated time points. At each caffeine concentration, 15 slugs were transferred for secondary tip formation and were monitored at 3 h, 4 h, 5 h, and 6 h of development. These values represent mean ± standard deviation.

To examine the kinetics of multiple tip formation, we stained *D. discoideum* amobae with neutral red and allowed them to develop as slugs which were transferred to a non-nutrient plates containing 5 mM caffeine. Neutral red stains the anterior prestalk region of the slug and during ectopic tip formation the staining gets redistributed from prestalk to prespore region of the slug after 1 h of development (Figure 1A). At 2 h of development, signs of slug fragmentation could be seen with slug cells aggregating locally as red coloured rings (Figure 1A) and this gets prominent after 4 hours. At 6 h of development, these local aggregates differentiate into secondary tips, (Figure 1A) and from each tip; a small fruiting body develops (Figure 1A). The number of tips increased as a function of time. The average number of tips observed at 3 h, 4 h, 5 h and 6 h of development at 5 mM concentration were 1.30 ± 0.5, 3.62 ± 1.06, 3.87 ± 1.12 and 3.87 ± 1.12, respectively (Figure 1B). We also investigated the kinetics of tip formation with different concentrations of caffeine. There is a correlation between the number of tips formed to the concentration of caffeine used (Figure 1B). The average number of tips formed at 2 mM, 3 mM, 4 mM, and 5 mM caffeine at 6 h of development are 1.30 ± 0.5, 3.62 ± 1.06, 3.87 ± 1.12 and 3.87 ± 1.12, respectively (Figure 1B). Thus, factors involved in repressing multiple tip formation may be inhibited by caffeine in a dose dependent and time dependent fashion.

### Caffeine induced multiple tip formation is conserved among slime molds

**Figure 2:**
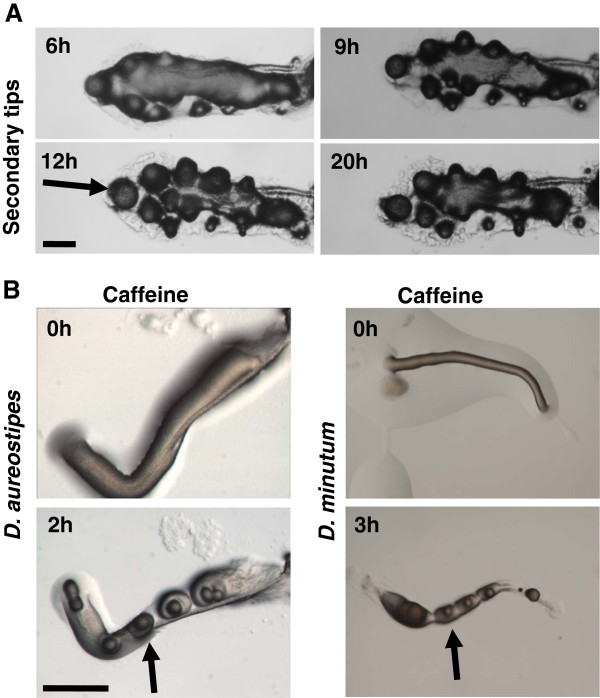
**Effect of caffeine on multiple tip formation in different Dictyostelids. A**) In *P. pallidum*, secondary tip formation was monitored at 6 h, 9 h, 12 h and 20 h of development. **B**) In *D. aureostipes *and *D. minutum*, ectopic tips were observed at 2 h and 3 h, respectively after transferring the slugs in plate containing 5 mM caffeine. Arrow indicates multiple tip formation at respective time intervals in different Dictyostelids. Scale bar = 200 μm.

Our earlier study demonstrated that certain regulatory mechanisms like aggregation is likely to be conserved in the entire cellular slime mold group irrespective of the chemoattractants they use [29]. To determine if secondary tip formation is also conserved among other cellular slime mold species, we chose one species from each group having distinct chemoattractant for their aggregation and monitored for multiple tip formation in the presence of caffeine. The group 2 species, *P. pallidum* formed secondary tip in the presence of caffeine similar to group 4 species *D. discoideum* (Figure 2A). In *P. pallidum* the tip formation was observed after 6 h of development and at 9 h of development it became pronounced which later differentiated into small fruiting bodies (Figure 2A). Caffeine induced multiple tip formation in group 1 species *D*. *aureostipes* and group 3 species *D. minutum,* were identical to secondary tips formed in group 2 species *P. pallidum* and group 4 species *D. discoideum* (Figure 2B). *D*. *aureostipes* and *D. minutum* were sensitive to caffeine action and showed prominent tip formation at 2 h and 3 h of development (Figure 2B), suggesting that the sensitivity to caffeine varies from species to species. Slime molds like *D*. *aureostipes* and *D. minutum* formed multiple tips earlier than other species implicating that caffeine sensitivity varies among Dictyostelids. Also, in species like *P. pallidum* many additional tips (Figure 2A) could be seen compared to other slime molds in the same concentration of caffeine suggesting that the sensitivity may be species specific. However, in spite of the variation with respect the time of origin of ectopic tips, this data strongly suggests that the mechanism regulating caffeine induced secondary tip formation may be conserved among the slime molds though they may use different chemoattractants.

### Effect of chemoattractants of one species on another for secondary tip effect

**Figure 3:**
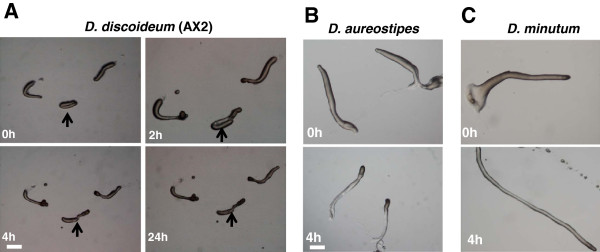
**Effect of extracellular cAMP on slugs of different *Dictyostelium *species.** The slug of *Dictyostelium *species were transformed on to a plate containing 0.2 mM cAMP and were observed at indicated time points. **A** and **B**) The slugs of *D. discoideum* and *D. aureostipes *did not culminate to fruiting bodies. **C**) The elongated slugs of *D. minutum *in the presence of 0.2 mM cAMP. Arrow in Figure 3A indicates the arrested development of AX2 slugs. Scale bar = 200 μm.

To check if chemoattractants of one species induces the secondary tip formation in another, we tested the effect of chemoattractants like cAMP and glorin on *D. minutum*, *D. aureostipes*; cAMP, folic acid and glorin in *D. discoideum*. In *D. discoideum* cAMP is a known chemoattractant [8]. *P. pallidum* uses glorin as a chemoattractant [13] and interestingly forms secondary tips at the migratory slug stage in the presence of cAMP [32]. None of these compounds such as 1.0 mM glorin, 5 mM folic acid and 0.2 mM cAMP were able to induce additional tips in the species tested but adding cAMP perturbed the slug morphology in all the species examined except *D. aureostipes* which collapsed and did not develop further (Figure 3). With cAMP, the slugs became slender and did not culminate at the concentration we tested (Figure 3). In the presence of cAMP, the slugs got thin in *D. discoideum* and did not elongate as much as we observed in other species like in *D. minutum.* Contrary to low cAMP levels promoting multiple tips, high cAMP levels favour slug elongation.

### Slugs with additional tips formed in the presence of caffeine showed reduced AcaA and Pde4 expression

**Figure 4:**
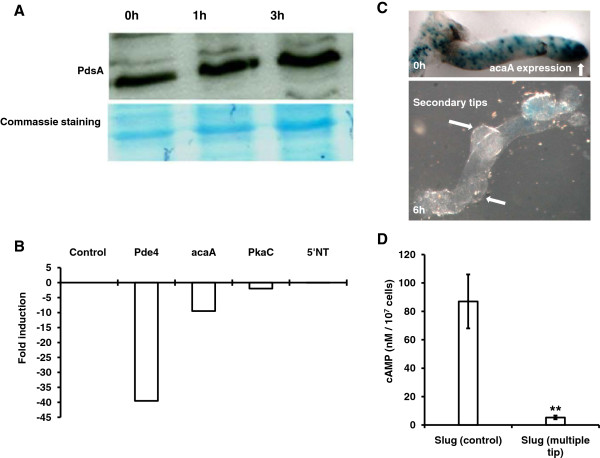
**Expression studies. ***D. discoideum *slugs (AX2) were used to perform these assays. **A**) Western blots: Expression of PdsA protein (AX2) at 0 h, 1 h and 3 h after transfer of slugs to 5 mM caffeine containing plates. **B**) Real time-PCR: Analysis of AcaA-mRNA, Pde4-mRNA, PkaC-mRNA and 5’NT-mRNA expression levels in multi tipped slugs at 6 h of development. **C**) AcaA-lacZ staining: Arrow indicates localization of Aca-*lacZ *in control slugs and the slugs having multiple tips (6 h of development). Reduced AcaA expression in the additional tips. **D**) cAMP quantification: graph shows absolute concentration of cAMP in the slugs formed from 1X 10^7^ cells. The cAMP levels were quantified in control and multi tipped slugs as mentioned in material and methods. These values represent mean ± standard deviation from 4 independent samples (Student t-test, **P < 0.01).

Adenyl cyclase-A (AcaA) is known to express in the slug tip [33] and catalyzes the conversion of ATP to cAMP. cAMP phosphodiesterase (Pde4 and PdsA) hydrolyzes cAMP into 5’AMP thus establishing a gradient along the anterior-posterior axis in slugs. If expression of these enzymes gets reduced, cAMP levels will go down during multiple tip formation and the cAMP relay signal may also get weak perturbing the cAMP gradient. To ascertain the strength of cAMP relay across the slug we performed quantitative analysis to assay both adenyl cyclase-A (acaA) and cAMP phosphodiesterase (PdsA and Pde4) during additional tip formation. We performed Real-Time PCR for AcaA-mRNA and Pde4-mRNA expression and western blot for PdsA protein. The expression of PdsA protein did not change in slugs having multiple tips (Figure 4A). However, the expression of the AcaA-mRNA and Pde4-mRNA reduced by 9.5 and 39.5 folds, respectively (Figure 4B). Further, we monitored AcaA-*lacZ* expression during multiple tip formation. In control slugs, the expression of AcaA-*lacZ* was confined to the slug tip and during secondary tip formation there was no expression at all in the slug (Figure 4C). The levels of cAMP in control slugs was 87 ± 19.2 nm/10^7^ cells and in slug having additional tips it was 5.7 ± 1.2 nm/10^7^ cells (Figure 4D). During extra tip formation, there seems to be correspondence of reduced cAMP levels with decreased expression of AcaA-mRNA and AcaA-*lacZ*. Based on western blot analysis, PdsA levels appear to be constant throughout the slug stage in the presence of caffeine suggesting that this enzyme may not be a major determinant during multiple tip formation. Thus, during additional tip formation cAMP gradient gets erased, AcaA expression goes down, low cAMP is synthesized and expression of cAMP phosphodiesterase (Pde4) also gets reduced.

### Inhibition of cAMP phosphodiesterase (Pde4) by IBMX gives rise to secondary tips in slugs

**Figure 5:**
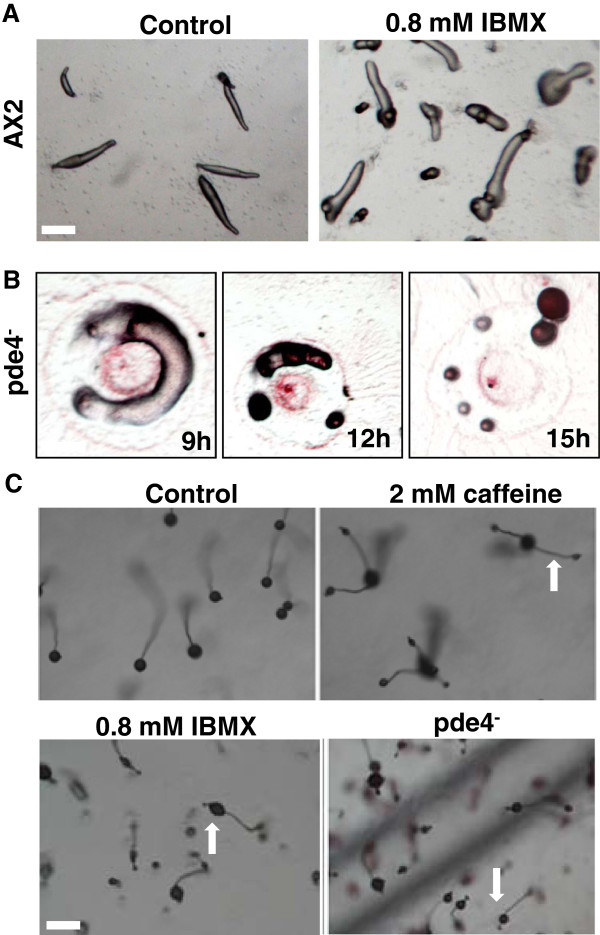
**Effect of IBMX (Iso-butyl methyl xanthine) on secondary tip formation. A**) Effect of 0.8 mM IBMX on secondary tip formation in AX2 slugs. AX2 slugs formed secondary tips after 12 h of development in the presence of 0.8 mM IBMX. **B**) The neutral red stained slug of Pde4 mutants showed secondary tips at 9 h and 12 h of development. At 15 h, every culminant transformed into fruiting bodies. **C**) Morphology of the fruiting bodies: Ectopic tips were observed in pde4 fruiting bodies and in the wild type fruiting bodies developed in the presence of 2 mM caffeine and IBMX as indicated with arrows. Scale bar = 200 μm.

Besides reduced cAMP levels, a perturbed cAMP gradient may also result in secondary tips. If extracellular cAMP phosphodiesterase (Pde4) is absent or its activity inhibited, then cAMP synthesized at the slug tip will not hydrolyze to AMP and therefore the gradient will not get established. Using a specific chemical inhibitor of extracellular cAMP phosphodiesterase (Pde4) [34,35] Iso butyl methyl xanthine (IBMX) we assayed for the formation of additional tips. First, we checked for secondary tip formation from slugs developed in the presence of 0.8 mM IBMX. A large fraction of the slugs had only one additional tip and rarely two tips (Figure 5A). In the presence of IBMX, secondary tips were seen after 12 hours of the development and mostly with one extra tip (Figure 5A). Most of the secondary tips were at the posterior region, few at center and rarely at the anterior of the slug (Figure 5A).

Further, we monitored the development of *pde4*^*-*^ cells on non-nutrient agar plates. *pde4*^*-*^ mound breaks and form tips after 9 hours of development (Figure 5B) and from each tip a small slug develops culminating to a fruiting body (Figure 5C). The fruiting body phenotype of Pde4 mutants was identical to the fruiting bodies formed in the presence of caffeine (Figure 5C).

However ectopic tips in the wild type fruiting bodies (AX2) developed in caffeine containing plates were prominent than the ones developed in the presence of IBMX (Figure 5C). Thus our experiment suggests that a stable cAMP gradient in the slug is required for normal development and its disruption leads to secondary tip formation.

### Mechanism regulating tip formation is similar in other slime molds

**Figure 6:**
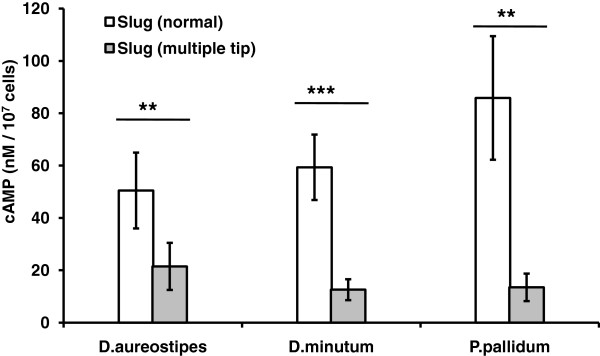
**cAMP concentration in secondary tipped slugs in different slime molds species. **cAMP levels were quantified in normal and multiple tipped slugs of *D. aureostipes*, *D. minutum*, and *P. pallidum *as mentioned in material and methods. Graph indicates significant reduction in cAMP levels in multi tipped slugs. Student t-test was performed to check the significance of the obtained values. These values represent mean ± standard deviation (Student t-test, ***P < 0.001, **P < 0.01).

cAMP is known to be a morphogenetic regulator in slime molds [8,9]. As shown in (Figure 4 and 5), during multiple tip formation, the cAMP gradient is disrupted and cAMP levels go down. We quantified cAMP concentration in slime molds from other groups such as *D. aureostipes* (group-1), *D. minutum* (group-3) and *P. pallidum* (group-2). Slugs with additional tips show reduced cAMP levels in all the species tested (Figure 6). The cAMP concentration in control slugs of *D. aureostipes*, *D. minutum*, *P. pallidum* were 50.5 ± 21.5, 59.37 ± 12.6 and 85.87 ± 13.5 nm/10^7^ cells, respectively. However in slugs with multiple tips, the cAMP concentration reduces drastically to 21.5 ± 9.0, 12.6 ± 4.0 and 13.5 ± 5.25 nm/10^7^ cells in *D. aureostipes*, *D. minutum*, *P. pallidum* respectively (Figure 6). These results suggest that in all the slime molds species, a perturbed cAMP level or a gradient or its signaling are the determining factors for the origin of the multiple tips.

### Impaired cell movement during multiple tip formation

**Figure 7:**
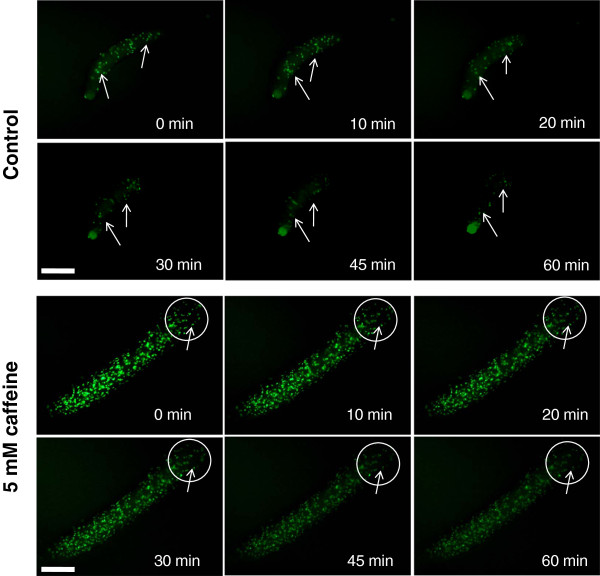
**Cell movement in slugs during secondary tip formation. **AX2 cells were labeled with a fluorescent dye (9 μM CFSE- Carboxyfluorescein succinimidyl ester for 30 minutes at 22°C in shaking conditions in dark). After 30 minutes, the cells were washed thrice with phosphate buffer and mixed with unlabeled cells in 1:49 ratio (CFSE-AX2: AX2). The slugs formed from a mixed population were transferred to a non-nutrient agar plate with or without 5 mM caffeine. Soon after the slug transfer, the movement of CFSE labeled cells was monitored by taking pictures using a fluorescence microscope at the indicated time point. Arrow highlights the cell movement within the slug. Scale bar = 200 μm.

Cell sorting in *Dictyostelium* is consequence of coordination between chemotactic cell movement and cAMP relay kinetics [27]. To determine if impaired slug movement in the presence of caffeine is due to asynchronous or altered chemotactic cell movement within the slug, we stained AX2 cells with a fluorescent dye Carboxyfluorescein succinimidyl ester (CFSE) and mixed them in the proportion of 1:49 to unlabeled cells and monitored the cell movement thereafter. We ensured all the cells were labeled and this dye is not known to leak out also. These CFSE labeled slugs were transferred onto non nutrient agar plates containing caffeine and in controls, plain buffered agar was used (Figure 7). Soon after the transfer to 5 mM caffeine containing plates, there was a highly restricted cell movement within the slug and the normal back to front movement was not seen (Additional file 1: Movie 1A and Additional file 2: Movie 1B). As caffeine restricted cell movement within the slug, the possibility of long range cell sorting could be ruled out and localized transdifferentiation of slug cells could be the cause for multiple tip formation.

### Cell sorting during multiple tip formation

**Figure 8:**
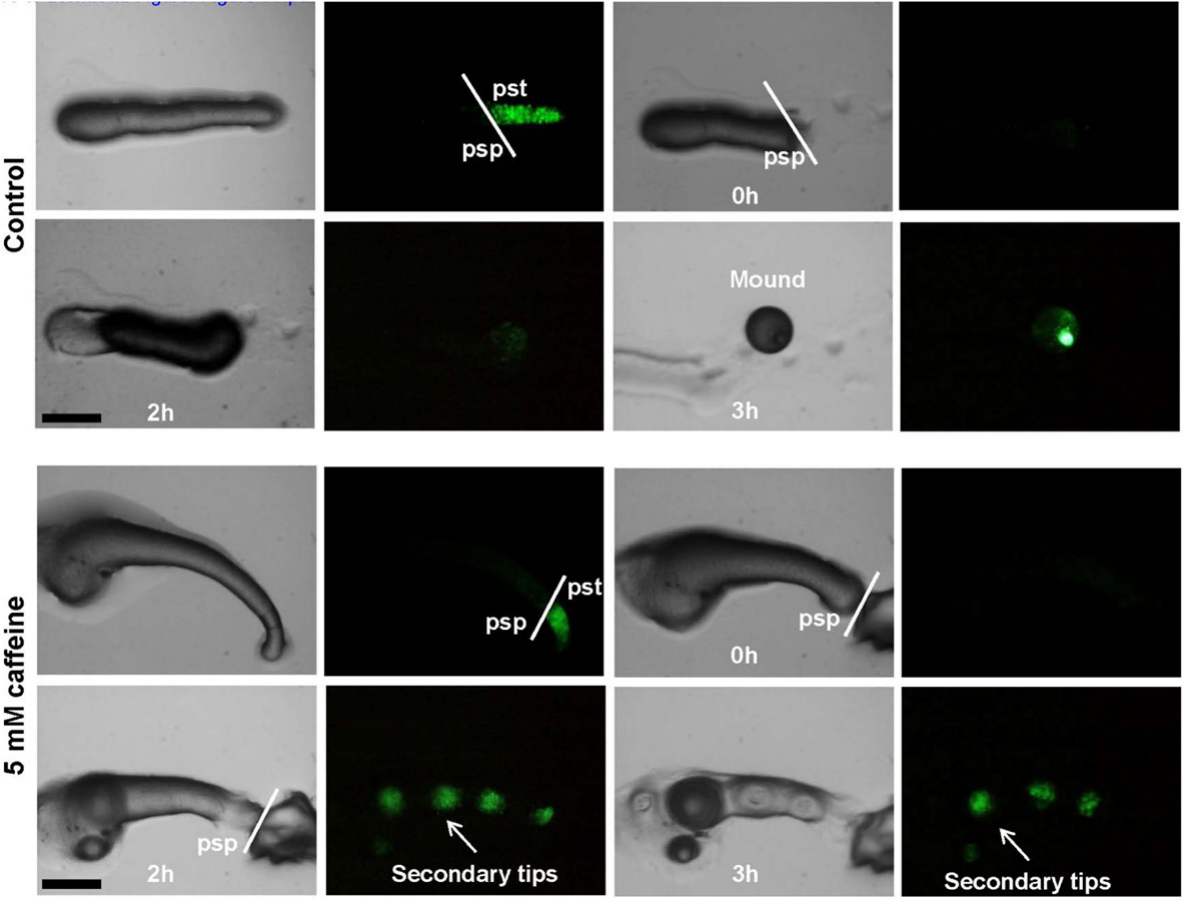
**Cell sorting during secondary tip formation. **The ecmA expressing part of slug (AX2-ecmA-GFP) was removed using a coverslip under an epi-fluorescent stereo zoom microscope. ecmA expression in control slugs (non-nutrient agar plate without caffeine) and those in caffeine containing plates was monitored at indicated time points. The arrow shows ecmA-GFP expression in the prespore region in prestalk ablated slug. Scale bar = 200 μm.

To test if the cells in slug transdifferentiate without extensive cell movement during multiple tip formation, we monitored the differential expression in slugs expressed a prestalk marker (ecmA-GFP) in the presence of 5 mM caffeine. The fluorescent ecmA-GFP expressing region of the slug was surgically removed and thereafter we checked the expression patterns during multiple tip formation. Faint fluorescent patch of expressing ecmA-GFP was observed 2 hours after removal (Figure 8) and the fluorescent intensity of the patch increased with time as tips became more prominent. In controls, the slug culminated normally and the ecmA expression was seen in the slug anterior region (Figure 8). In the presence of caffeine, there was highly restricted cell movement in the slug. If prestalk ecmA expression is observed in the prespore (psp) region, it would suggest localized transdiffrentiation of ‘psp’ cells to ‘pst’ cells during additional tip formation.

### The fate and proportion of the cell types in secondary tips

**Figure 9:**
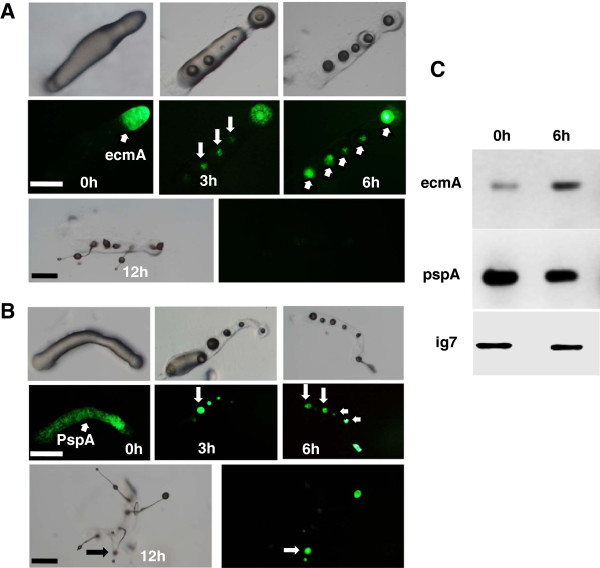
**The fate of cell types in secondary tips. A**) The fate of ecmA (AX2-ecmA-GFP) during multiple tip formation at 3 h, 6 h and 12 h of development. Confined ecmA-GFP expression in the secondary tips. Scale bar = 200 μm. **B**) Fate of PspA (AX2-pspA-GFP) during multiple tip formation. The expression of PspA-GFP was monitored by taking pictures at 3 h, 6 h and 12 h of development. PspA-GFP expression in secondary tips and in fruiting bodies. Scale bar = 200 μm. The arrow indicates ecmA-GFP and pspA-GFP expression in the secondary tips. **C**) Semi-quantitative expression analysis (RT-PCR) of the ecmA and PspA mRNA in slugs having secondary tips. For comparative analysis of the transcript levels of ecmA and PspA, a semi-quantitative expression analysis was performed. Total RNA was extracted and cDNA was synthesized as described in material and methods. Equal amount of cDNA were used for all experiments and PCR was carried out for 35 cycles. Ig7 gene was used as an internal control. RT-PCR was performed from three independent samples. ecmA expression increased by three fold while PspA expression decreased by 50%. Primer sequences are mentioned in Table 2.

The proportion of prespore to prestalk cells in the slug is tightly controlled in slime molds [2] but in certain conditions it could vary. To determine if either prestalk or prespore or both cell types contribute towards supernumerary tip formation, we chose cell type specific markers, ecmA, the prestalk marker and pspA, the prespore marker and monitored their spatial pattern periodically by recording the GFP reporter activity. During secondary tip formation, the ecmA expression can be seen as several spots with bright fluorescence in the prespore region of the slug and stalk of the secondary fruiting bodies (Figure 9A). PspA expression was also seen in secondary tips and spore head of the fruiting bodies (Figure 9B). Based on ecmA and PspA expression, both the prestalk and prespore cell types may be contributing to multiple tip formation. However the proportion of the cell types might be different during ectopic tip initiation. Hence, we checked ecmA-mRNA and PspA-mRNA levels by semi-quantitative PCR at 6 h of multiple tip formation. ecmA expression increased by three fold while PspA expression decreased by 50% (Figure 9C). Semi-quantitative PCR analysis at the multiple tip stage suggests that the proportion of prestalk cells in secondary tips are high compared to the prespore fraction indicating that there is a transdiffrentiation of prespore to prestalk cells resulting in increased proportion of ecmA.

**Table 2:** List of primers used in semi quantitative PCR

**Gene**	**Forward primer**	**Reverse primer**
pspA	CATTGGCCAATCAAAATCCAG	ACAACAGTTGAAGCAGAACC
ecmA	CCAATTAGCTGTCCAAAACC	GCAATCACCTTTACCTCCTG
IG7	TTACATTTATTAGACCCGAAACCAAGCG	TTCCCTTTAGACCTATGGACCTTAGCG

## Discussion

In *Dictyostelium*, the differentiation of cell types and its fate is regulated by morphogens which are DIF (Differentiation Inducing Factor), cAMP, ammonia and adenosine [2]. In *D. discoideum*, cAMP acts as a chemoattractant as well as regulating cell differentiation [8]. However, in other slime mold species like *D. minutum*, *P. pallidum* and *D. aureostipes,* cAMP is involved in cell differentiation only and not chemotaxis [7,9,13]. The slug tip acts as an organiser and regulates the volume and shape [14]. In *D. discoideum*, adenosine is known to prevent competing tip formation by favouring tip dominance [14]. In the branched slime mold *Polysphondylium* the primary tip is known to inhibit secondary tip formation [36] and surgical removal of the apical tip result in secondary tip emergence suggesting the dominance of the apical tip. During its development *Polysphondylium* goes through a spontaneous spherical to radial symmetry breaking event and during this transition many tip arise around the equator of a spherical mass of cells equidistant from each other suggesting that that one tip inhibits the other [37]. Probably this lateral inhibiton in *Polysphondylium* is akin to caffeine induced multiple tip effect. Multiple tip phenotype induced by caffeine was observed in all the slime molds species we investigated suggesting a conserved mechanism regulating secondary tip formation in slime molds. The formation of secondary tips is controlled by relayed cAMP signal strength and suppression of tip dominance [17]. In this study, we examined the mechanism that regulates multiple tip formation in the presence of caffeine. Both in the previous report [17] and in this work, millimolar concentrations of caffeine have been used to generate multiple tip effect. Being soil amoebae, slime molds have to encounter a variety of environments and may have efficient ABC transporters to thrive and hence all the effects can be observed only at millimolar concentrations [38].

Few known mechanisms of caffeine action include inhibition of cyclic nucleotide phosphodiesterase, competitive inhibition of adenosine receptors, inhibition of ryanodine receptors [39] and inactivation of Target of Rapamycin complex (TOR complex) [40]. Adenosine receptors are not known to be present in the *Dictyostelium* genome and hence caffeine could possibly target cAMP phosphodiesterases (PdsA and Pde4) or ryanodine receptor. It is not known if caffeine targets ryanodine receptors in *Dictyostelium* but caffeine does not affect PdsA levels. It is likely that caffeine by impairing cAMP relay and altering intracellular calcium levels cause pleiotropic effect on signaling, motility and gene expression all leading to multiple tip formation.

Theophylline (1, 3 dimethyl xanthine), theobromine (3, 7 dimethyl xanthine) and paraxanthine (1, 7 dimethyl xanthine) are caffeine analogs, sharing a common structural xanthine backbone [41,42]. These compounds are known to have specific selectivity towards different targets like adenosine receptors, and calcium channels [41,42]. Caffeine being highly lipophilic (trimethyl xanthine) than theobromine or theophylline (dimethyl xanthine) may be why it is most effective, suggesting an intracellular function of caffeine possibly through opening calcium stores. In the presence of caffeine analogs and adenosine, the slugs continued to move which could be tracked by a trail that it leaves behind (Additional file 3: Figure S1). When slugs were transferred to a plate containing 100 μM A23187 + 1 mM CaCl_2_ it did not result in secondary tip formation (data not shown) the slugs rounded up similar to a mound seen in controls (without drug). Similarly, when slugs were transferred to a plate containing 5 mM EGTA, there was arrested development (data not shown). Studies with caffeine analogs and supplementing calcium with a specific ionophore did not result in multiple tip formation suggesting that an increase of cytosolic calcium alone is not responsible for additional tip effect and a caffeine specific pool of calcium reservoirs is activated during additional tip formation.

During post aggregate stages of development, the tip of the slug continues to be a source of cAMP signalling [43]. Adenyl cyclases catalyse the conversion ATP into cAMP. During multiple tip formation, cAMP levels decrease as a consequence of reduced adenyl cyclase-A expression and its activity. Adenyl cyclase-A is regulated by the activity of cytosolic regulator of adenyl cyclase **(**CRAC) and Target of Rapamycin complex-2 (TORC2) [44]. In yeast, caffeine acts on TOR1 unit of TORC1 complex and inactivates it [45]. However, in *Dictyostelium* TOR is a part of both TORC1 and TORC2 so it is likely that caffeine can inactivate both TORC1 and TORC2. TORC2 regulates chemotaxis and multicellular organization by monitoring the expression levels of adenyl cyclase-A (acaA) and cAMP signal relay. The reduced expression of adenyl cyclses and decreased cAMP levels in slugs with additional tips (in the presence of caffeine) can be due to impaired activity of TORC2. However, in the presence of rapamycin, we did not observe multiple tips in the slugs (data not shown). It is not known if rapamycin affects pde4 levels. Also the specific calcium pool mobilized by caffeine may not be responding to rapamycin though both could impair TORC2 activity. Though caffeine and rapamycin are known to induce identical set of genes in yeast [46], structurally they are different and caffeine effect is pleiotropic. So, caffeine besides affecting TORC2 and decreasing cAMP levels can interact with other proteins such as cAMP phosphodiesterase (PdsA and Pde4) which are involved in maintaining cAMP gradient and that could also significantly contribute to multiple tip formation and all these effects may not happen with rapamycin alone.

*In vitro* experiments suggest that PdsA levels are not inhibited by caffeine and so it is likely that Pde4 is a key component responsible for maintaining gradients of cAMP as well as adenosine. cAMP phosphodiesterase (Pde4) hydrolyses cAMP into AMP thus generating cAMP gradient from anterior to the posterior region of the slug. Overproduction of the phosphodiesterase Pde4 is known to arrest development at the tight mound stage, prior to tip formation. During multiple tip formation, cAMP phosphodiesterase (Pde4) levels go down and adenosine levels and cAMP gradient also gets perturbed. Inhibition of cAMP phosphodiesterase (Pde4) activity with iso butyl methyl xanthine (IBMX) results in secondary tips in slugs and fruiting bodies. Though application of IBMX, a proven inhibitor of Pde4 gives rise to multiple tips, the phenotype is prominent with caffeine. Further, many pleiotropic pathways converge to give multiple tip effect whereas IBMX effect may be specific to Pde4 alone.

Adenosine is the breakdown product of cyclic AMP, and known to act as an antagonist to cyclic AMP, providing an intrinsic negative feedback loop [14,47]. Adenosine is known to inhibit competitive tip formation by tip dominance. Multiple tips formed in the presence of caffeine can also be due to the suppression of tip dominance. Tips formed at the anterior of the slug are smaller compared to the ones at the posterior suggesting that anterior end exert maximum inhibition of tip dominance and it gets weaker to the posterior of slug. Secondary tip formation in *Polysphondylium* in the presence of extracellular cAMP suggests the disruption of cAMP gradient leading to tip formation elsewhere in the slug [32]. PkaC is known to regulate cAMP relay signal and cells lacking it are impaired in aggregation [22]. It is not clear if a 1.6 fold decrease in the expression of PkaC would significantly contribute to multiple tip effect as real time PCR data showed no significant changes in the expression of PkaC between slugs having multiple tips and control (Figure 4). We propose that reduced cAMP levels, suppression of tip dominance and altered cAMP gradient, together cause secondary tip formation in slugs.

It is known that a positional signal in isolated prestalk or prespore part induces localized transdifferentiation of the appropriate cell type to form the prestalk and prespore pattern. During secondary tip formation, we observed the prestalk marker EcmA-GFP expression in the prespore region indicating a position–dependent mechanism of regulation. Cells in the prespore region of the slug move in periodic fashion with a speed of 20 μm / minute [17]. If the slug tip is removed it impairs cell movement in the prespore region and all the prespore cells form a mound. ALCs scattered along the slug length and prespore cells re-differentiate to prestalk cells until a proportioned slug is formed [48]. Cells within the caffeine treated slugs do not migrate far and undergo transdifferentiation, forming local aggregation centres resulting in secondary tips. ALCs scattered in the prespore region are likely to initiate the first organising center and additional prespore cells are recruited along with ALCs to form multiple tips [17]. Prestalk EcmA marker expression analysis during secondary tip formation suggests cell movement is severely impaired during ectopic tip formation. If there is no cell movement, then transdifferentiation could be the reason for multiple tip effect, which is what we observed. Semi-quatititative analysis of ecmA and pspA expression showed that prestalk and prespore cells are disproportionate during secondary tip emergence. The redifferentiation of prespore and prestalk cells is controlled by cAMP and DIF-1 (Diffrentiation inducing factor) in a combinatorial manner. In the presence of DIF-1, spore differentiation is inhibited and the cells become stalk instead [4] so the stalk cell diffrentiation in prespore part can be modulated by DIF-1. cAMP is known to induce prespore cell differentiation [2] and thus the expression of prespore marker in prestalk part might be carried out by cAMP. The surface sheath of pseudoplasmodium also plays an important role regulating spore differentiation [49]. The low molecular weight, diffusible effectors mainly NH_3_ and cAMP determines the thickness of the sheath [49]. The sheath covering the posterior prespore region is thicker compared to the slug anterior and this may also play a critical role in establishing gradients of cAMP [49]. During secondary tip formation, the levels of these effector molecules in the posterior region of the slug may decrease and it is not sure if the thickness of the sheath also reduces. The thinner sheath of the pseudoplasmodium does not favour spore cell differentiation and hence cells sort out to the prestalk and form secondary tips in varied prestalk/prespore cell proportions.

Soon after transferring the slugs to caffeine containing plates, the cell migration stops within the slug and impaired cell movement alone may not be responsible for giving the multi-tipped slug. The multiple tip effect gets severe with time suggesting that the phenotype that we observe depends on changes in gene expression also. Prolonged observation with intense beam of light for microscopic observations prevented multiple tip formation in the presence of caffeine (data not shown).

Few examples of mutants with additional tips include the ones carrying genetic lesions in 5’ nucleotidase and TipA and TipB mutants [50,51]. The latter two have defects in sorting of prestalk cells which also show a multiple tip phenotype in the mound stage c. Treating these Tip mutants with caffeine failed to show any change in their phenotype and adenosine failed to rescue these defects either. (Additional file 4: Figure S2). Caffeine besides inducing multiple tips in *P. pallidum* slugs, affects fruiting body branching with tertiary branches coming out of the secondary stalks (Additional file 5: Figure S3).

The novel finding of this work includes 1. Conserved action of caffeine in inducing multiple tips in different slime mold species. 2. In all species examined, caffeine perturbs cAMP signaling, its levels and gradient within the slug possibly favoring multiple tip formation. 3. The origin of multiple organizing centers is a caffeine specific effect and its analogs fail to give ectopic tips. 4. During ectopic tip formation, there is a highly restricted cell movement and trans-differentiation of prespore to prestalk cell types takes place within the slug.

## Conclusion

The effect of caffeine was monitored on different slime mold species for multiple tip formation and in this study, we show that certain mechanisms of multiple tip formation seems to be conserved among distantly related slime molds and factors/mechanism regulating tips formation is also similar in other slime molds. The multiple tip formation is specific to the presence of caffeine. Caffeine reduces cAMP levels in slugs altering its gradient and relay thereby inducing tips elsewhere in the slug. Our work also suggests that cAMP playing a critical role in other slime molds during later stages of development.

## Competing interests

All the authors declare that no competing interests exist.

## Authors’ contributions

PJ and RB designed the experiments. PJ performed most of the experiments and analyzed all the data. SPS, PA and RA performed the qRT-PCR, caffeine analog and cell sorting experiments. PJ and RB wrote the manuscript and all authors read and approved the final manuscript.

## Funding

This work was supported by Department of Biotechnology (DBT), Council of Scientific and Industrial Research (CSIR), New Delhi, Government of India.

## Supplementary Material

Additional file 1 **Movie 1A. **Cell movement in control slug. Click here for file

Additional file 2 **Movie 1B. **Cell movement in slug in the presence of caffeine (during multiple tip formation). Click here for file

Additional file 3 **Figure S1. **Effect of caffeine analogs (5 mM) on secondary tip formation. Theophylline, paraxanthine, theobromine and adenosine were checked for the secondary tip formation in slugs *D. discoideum*. Slugs in the presence of these compounds culminated except caffeine which induced secondary tip formation. Scale bar = 200 μm. Click here for file

Additional file 4 **Figure S2. **Effect on caffeine and adenosine on TipA and TipB mutants at mound stage. Both compounds showed no response in promoting multiple tips. Scale bar = 200 μm. (PDF 101 kb) Click here for file

Additional file 5 **Figure S3. **Effect of 1 mM caffeine on fruiting bodies of *P. pallidum*. Scale bar=1000 μm. Click here for file
