# Supplementary material for: Regulation of multiple tip formation by caffeine in cellular slime molds
Source: BMC Dev Biol. 2012 Aug 28;12:26. doi: 10.1186/1471-213X-12-26 (PMC3488011; doi:10.1186/1471-213X-12-26)
Supplement: Additional file 3 — Figure S1. Effect of caffeine analogs (5 mM) on secondary tip formation. Theophylline, paraxanthine, theobromine and adenosine were checked for the secondary tip formation in slugs D. discoideum. Slugs in the presence of these compounds culminated except caffeine which induced secondary tip formation. Scale bar = 200 μm. [file 1471-213X-12-26-S3.pdf]

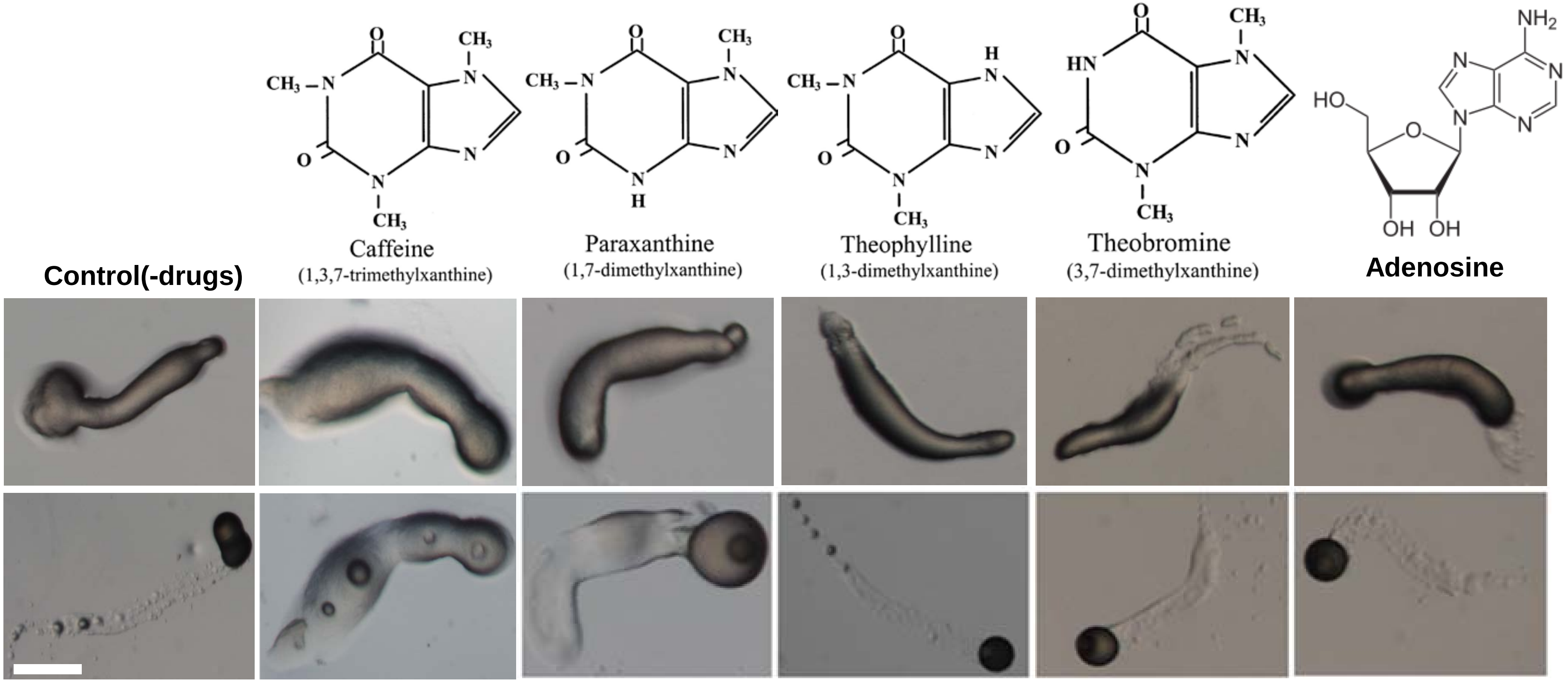

Control(-drugs)
Caffeine
(1,3,7-trimethylxanthine)
Paraxanthine
(1,7-dimethylxanthine)
Theophylline
(1,3-dimethylxanthine)
Theobromine
(3,7-dimethylxanthine)
Adenosine
