# Supplementary figures and images for: Regulation of multiple tip formation by caffeine in cellular slime molds
Source: BMC Dev Biol. 2012 Aug 28;12:26. doi: 10.1186/1471-213X-12-26 (PMC3488011; doi:10.1186/1471-213X-12-26)

**Control**

**2 mM caffeine**

**2 mM adenosine**

**tipA<sup>-</sup>**

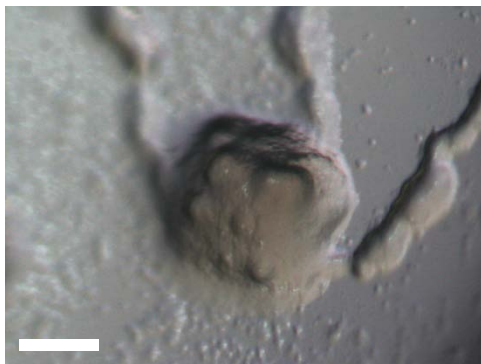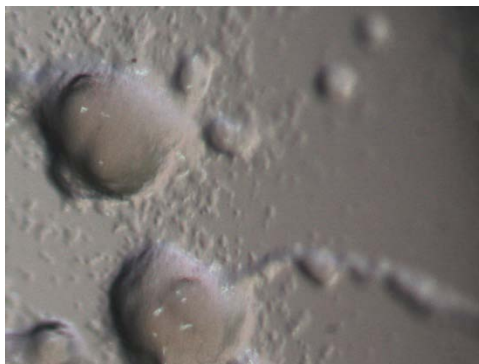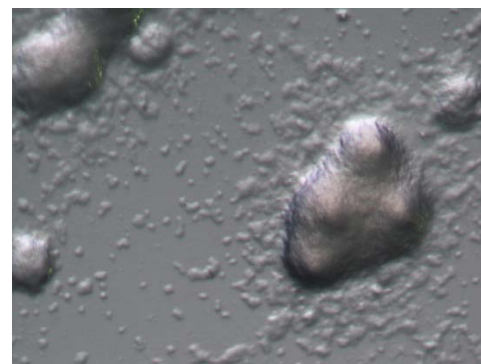

**tipB<sup>-</sup>**

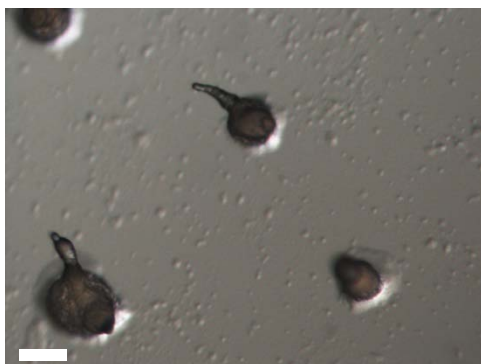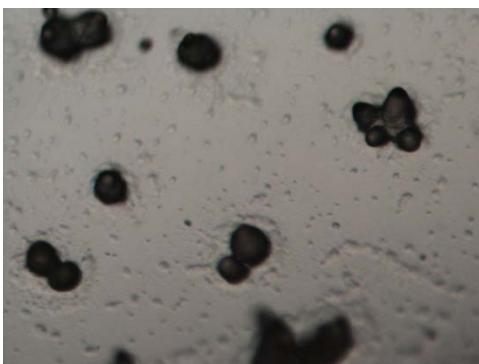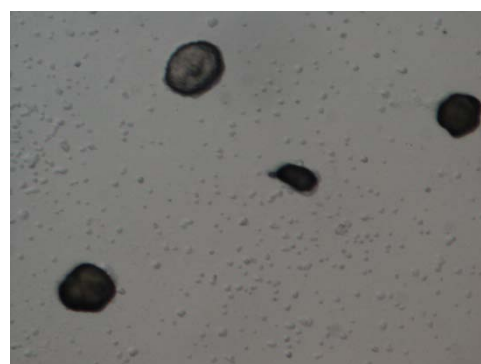

Supplement: Additional file 4 — Figure S2. Effect on caffeine and adenosine on TipA and TipB mutants at mound stage. Both compounds showed no response in promoting multiple tips. Scale bar = 200 μm. (PDF 101 kb) [file 1471-213X-12-26-S4.pdf]

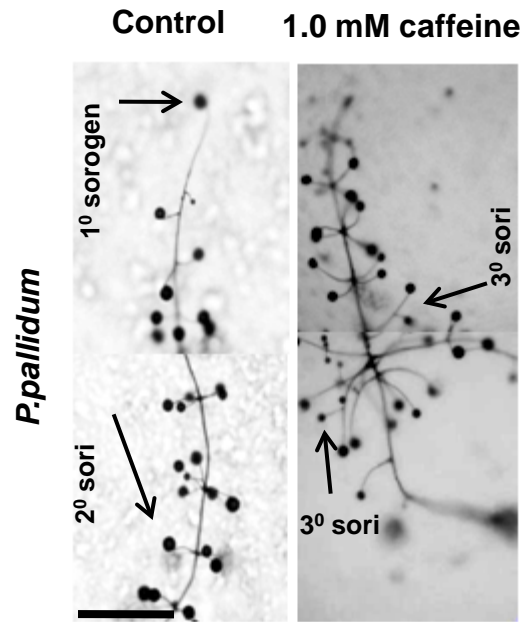

Supplement: Additional file 5 — Figure S3. Effect of 1 mM caffeine on fruiting bodies of P. pallidum. Scale bar=1000 μm. [file 1471-213X-12-26-S5.pdf]
